# Pleistocene climate and geomorphology drive the evolution and phylogeographic pattern of *Triplophysa robusta* (Kessler, 1876)

**DOI:** 10.3389/fgene.2022.955382

**Published:** 2022-09-12

**Authors:** Hui Zhong, Yaxian Sun, Huihui Wu, Shengnan Li, Zhongyuan Shen, Conghui Yang, Ming Wen, Peng Chen, Qianhong Gu

**Affiliations:** ^1^ The State Key Laboratory of Developmental Biology of Freshwater Fish, College of Life Sciences, Hunan Normal University, Changsha, China; ^2^ College of Fisheries, Engineering Technology Research Center of Henan Province for Aquatic Animal Cultivation, Henan Normal University, Xinxiang, China; ^3^ Xinjiang Uygur Autonomous Region Fishery Research Institute, Urumchi, China

**Keywords:** Qinling mountains, vicariance event, past climate change, phylogeographic pattern, T. robusta

## Abstract

Montane systems provide excellent opportunities to study the rapid radiation influenced by geological and climatic processes. We assessed the role of Pleistocene climatic oscillations and mountain building on the evolution history of *Triplophysa robusta*, a cold-adapted species restricted to high elevations in China. We found seven differentiated sublineages of *T. robusta*, which were established during the Mid Pleistocene 0.87–0.61 Mya. The species distribution modeling (SDM) showed an expansion of *T. robusta* during the Last Glacial Maximum (LGM) and a considerable retraction during the Last Interglacial (LIG). The deep divergence between Clade I distributed in Qinling Mountains and Clade II in Northeastern Qinghai-Tibet Plateau (QTP) was mainly the result of a vicariance event caused by the rapid uplifting of Qinling Mountains during the Early Pleistocene. While the middling to high level of historical gene flow among different sublineages could be attributed to the dispersal events connected to the repetition of the glacial period during the Pleistocene. Our findings suggested that frequent range expansions and regressions due to Pleistocene glaciers likely have been crucial for driving the phylogeographic pattern of *T. robusta*. Finally, we urge a burning question in future conservation projection on the vulnerable cold-adapted species endemic to high elevations, as they would be negatively impacted by the recent rapid climate warming.

## 1 Introduction

The process of population isolation and differentiation, the core of micro-evolution, is one of the crucial steps for deciphering speciation, which has long fascinated evolutionary biologists (Rice and Hostert, 1993; Wang et al., 2021). The Earth’s complex topographical history often generates opportunities for population isolation and differentiation (Yan et al., 2010; Hou et al., 2014; Wang et al., 2021), especially for montane species. Mountain-building is often associated with tectonic forces, promoting population divergence through vicariance (Hoorn et al., 2010; Smith et al., 2014; Simões et al., 2016; Schramm et al., 2021). However, previous studies suggested that mountain building might not be regarded as the sole driver in the diversification of montane species (Kong et al., 2022). Climate change is also considered a major driver of vicariant speciation and intraspecific divergence and animal radiations (Wang et al., 2012; de Oliveira et al., 2021). Particularly, climatic changes associated with Pleistocene glacial cycles are believed to have caused montane species to shift, expand, or contract along latitudinal or elevational gradients (Wang et al., 2012). Populations adapted to different mountains may have experienced isolations due to the range contractions, or may have experienced connectivity as a result of range expansions following climate fluctuations and geological activity.

The drastic uplift of the Qinghai-Tibet Plateau (QTP) and the resulting Asian monsoons largely reshaped the landscape of local and adjacent regions (Clark et al., 2005; Zhang et al., 2012). For example, the Northeastward growth of the Tibetan Plateau since the late Cenozoic and the Northern Qinling Mountains have been strongly reactivated and uplifted. The Qinling Mountains rapidly uplifted during the Miocene-Quaternary (Dong et al., 2011; Liu et al., 2013; Meng, 2017), and extended more than 1,500 km from West to East in Central China. The diverse topography and varied habitats in Qinling Mountains come into being a biogeographical barrier for wild species (Yan et al., 2010; Huang et al., 2017; Wang et al., 2021). Both the Northeastern QTP and Qinling Mountains are important regions for the evolution of biota (Wang et al., 2012; Feng et al., 2019).

*Triplophysa* are endemic species in QTP and its adjacent area. It is believed that the origin and evolution of *Triplophysa* are related to the uplift of the QTP (Wang et al., 2016; Li et al., 2017). This group has been one major component of fish fauna on the QTP and the adjacent area and has evolved specific morphological and physiological adaptations to the low oxygen, low pressure, and cold conditions of the plateau (Wu et al., 2020). *T. robusta* is endemic to the Upper Yellow River and the upper reaches of the Jialing River (Feng et al., 2019). However, it was reported that *T. robusta* could be found in the upper reaches of the Yihe River and Luohe River, distributed in the Qinling Mountains, as well as the montane streams in the Southern Taihang Mountains (Wu et al., 2020). Therefore, *T. robusta* represents an ideal model to address the effects of past climatic and geological events on the intraspecific diversification among populations adjacent to and distant from QTP. Hence we used mitochondrial and nuclear genes to estimate the phylogeographic pattern and evolutionary history of *T. robusta*, and to evaluate the conservation status and strategy of this species.

## 2 Materials and methods

### 2.1 Samples and sequence data preparation

**FIGURE 1:**
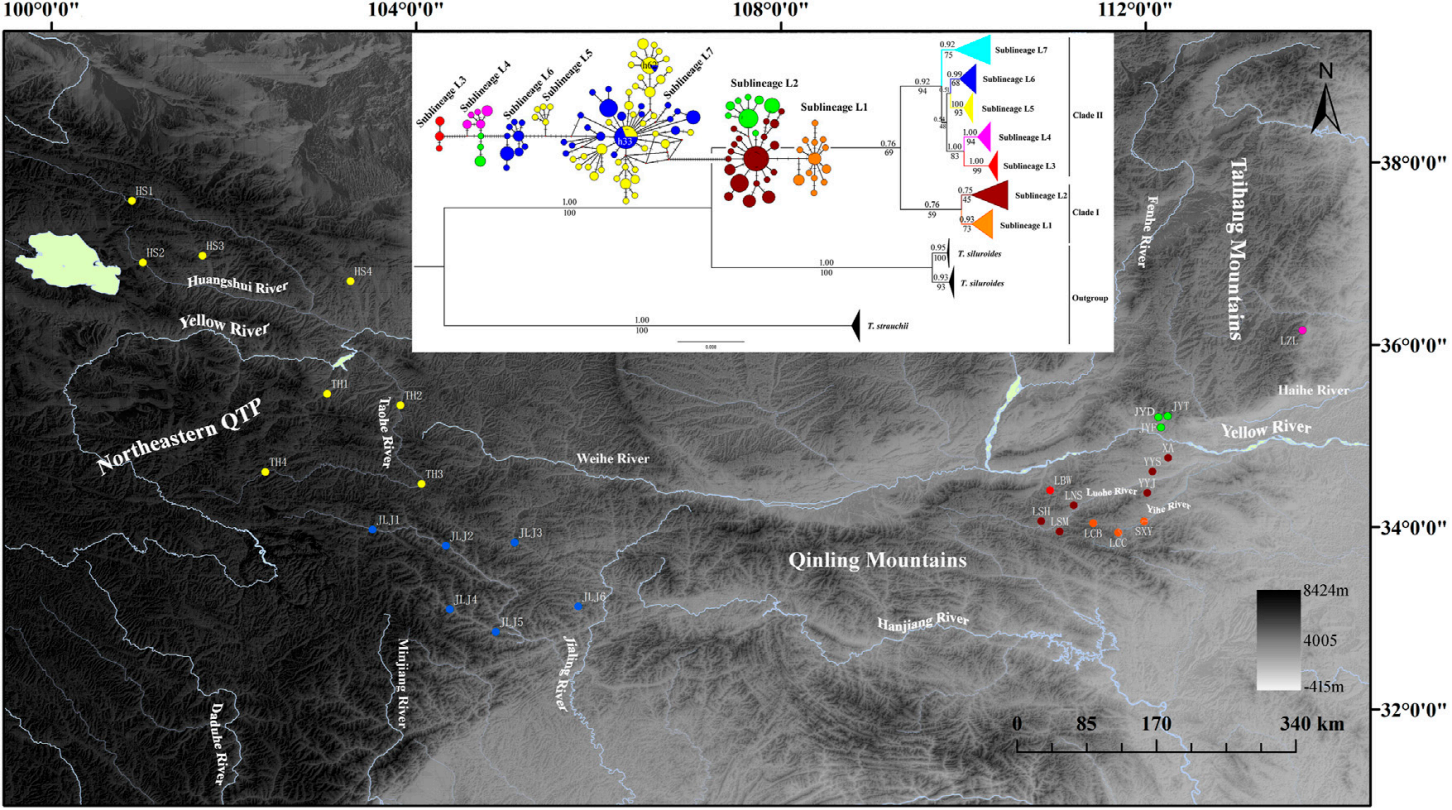
Map of sampling locations of *T. robusta*, localities are detailed in Table 1 according to Wu et al. (2020) and Feng et al. (2019), and the phylogenetic tree based on Bayesian inference (values on branches indicate bootstrap proportions from a maximum likelihood analysis and bayesian posterior probabilities), and the median-joining haplotype network based on 235 *cytb* sequences, the size of the circles represents haplotype frequency. Each connecting line represents a single nucleotide substitution, and each little short line represents a mutated position. Different river systems were represented in different colors, consistent with the colors of the haplotype.

Ten populations of *T. robusta* in the Eastern Qinling Mountains and four populations in the Southern Taihang Mountains (Figure 1; Table 1) were collected in 2017 and 2018. The genomic DNA was extracted and the nuclear genes rhodopsin (*RH1*) was amplified following Feng et al. (2019). We sequenced 42 novel *RH1* genes with three individuals in each population, and they have been deposited in GenBank (accession number, ON630917-ON630958). Three mitochondrial genes (16S rRNA, *16S*; cytochrome b, *cytb*, cytochrome oxidase I, *COI*, Supplementary Table S1) were obtained from previous studies (Feng et al., 2019; Wu et al., 2020). A total of 237 individuals from 28 localities across the major distribution of *T. robusta* were collected in China (Figure 1; Table 1). Besides, another 122 *RH1* sequences were also retrieved from the previous study (Feng et al., 2019), and a total of 164 *RH1* sequences were used to investigate the populations’ relationship among the Northeastern QTP, the Eastern Qinling Mountains, and the Southern Taihang Mountains.

**TABLE 1:** Detailed information for specimens included in this study and diversity indices for each population based on *cytb*.

Pop ID	N	Locations	Latitude	Longitude	Number of genes sequenced				Diversity indices		
					*16S*	*COI*	*cyt b*	*RH1*	S	Hd	π
HS1	13	Huangshui River	37.578	100.881	13	0	13	13	12	0.936	0.00317
HS2	8	Huangshui River	36.900	101.000	8	0	8	8	18	0.929	0.00501
HS3	3	Huangshui River	36.977	101.654	3	0	3	3	6	1.000	0.00351
HS4	7	Huangshui River	36.697	103.278	7	0	7	7	19	0.810	0.00643
TH1	10	Taohe River	35.460	103.022	10	0	10	10	19	1.000	0.00541
TH2	16	Taohe River	35.335	103.825	16	0	16	16	24	0.975	0.00448
TH3	7	Taohe River	34.473	104.057	7	0	7	7	16	0.929	0.00473
TH4	1	Taohe River	34.604	102.344	1	0	1	1	NA	NA	NA
JLJ1	17	Jialing River	33.973	103.522	17	0	17	17	16	0.926	0.00501
JLJ2	5	Jialing River	33.795	104.325	5	0	5	5	18	1.000	0.00825
JLJ3	3	Jialing River	33.830	105.080	3	0	3	3	12	1.000	0.00702
JLJ4	3	Jialing River	33.099	104.367	3	0	3	3	5	1.000	0.00292
JLJ5	23	Jialing River	32.849	104.872	23	0	23	23	8	0.818	0.00139
JLJ6	6	Jialing River	33.128	105.776	6	0	6	6	14	0.933	0.00427
LZL	9	Lushui River	36.157	113.718	0	9	9	3	4	0.861	0.00120
LBW	6	Dongjian River	34.403	110.952	0	5	5	3	25	0.867	0.00786
LCB	4	Yihe River	34.043	111.423	0	4	4	3	4	0.833	0.00195
LCC	9	Yihe River	33.939	111.697	0	9	9	3	11	0.889	0.00320
SXY	6	Yihe River	34.064	111.980	0	6	6	3	7	0.933	0.00246
LNS	9	Luohe River	34.240	111.211	0	9	9	3	10	0.917	0.00218
LSH	3	Luohe River	34.064	110.852	0	3	3	3	3	0.667	0.00180
LSM	9	Luohe River	33.951	111.055	0	9	9	3	12	0.889	0.00360
XA	16	Luohe River	34.758	112.245	0	16	16	3	4	0.725	0.00116
YYJ	5	Luohe River	34.376	112.016	0	5	5	3	2	0.400	0.00072
YYS	9	Luohe River	34.610	112.073	0	9	9	3	0	0.000	0.00000
JYD	11	Dongyang River	35.203	112.139	0	11	11	3	15	0.764	0.00517
JYF	7	Fengshi River	35.093	112.167	0	7	7	3	13	0.810	0.00523
JYT	12	Tieshan River	35.215	112.240	0	12	10	3	49	0.933	0.02100
Total	237				122	114	234	164	153	0.985	0.01846

N means the number of individuals in each population, S indicates the number of polymorphic sites, Hd indicates haplotype diversity, π indicates nucleotide diversity, NA, means absent.

### 2.2 Phylogenetic analysis

All sequences were aligned by using MAFFT implemented in PhyloSuite v1.2.2. We tested the homogeneity of the combined genes (*COI* + *cytb* + *16S* + *RH1*) in *PAUP and the *p*-value was 0.074 (>0.05). Consequently, the aligned nucleotide sequences *cytb*, *COI*, *16S,* and *RH1* were concatenated to construct the phylogenetic tree by using the maximum likelihood (ML) method implemented in RAxML v8.2.4 with 1,000 bootstrap replicates. Each gene was treated as unlinked since it is not required to have the same individuals analyzed for every genetic marker if partitions are considered as unlinked (Sota and Vogler, 2001; Husemann et al., 2013; Gu et al., 2022). Bayesian inferences (BI) analysis was performed in MrBayes v3.2.6 using the Markov Chain Monte Carlo method with 10 million generations and sampling trees every 1,000 generations. The first 25% of trees were discarded as burn-in with the remaining trees being used for generating a consensus tree. The species of *Triplophysa siluroides* and *Triplophysa strauchii* (Supplementary Table S1) were used as outgroups. The final trees were visualized in FIGTREE v1.4.4.

To further visualize haplotype diversity and distribution of *T. robusta*, we generated a haplotype network using 235 *cytb* sequences and colored each haplotype by the geographic region from which river system it was collected (Table 1). The haplotype networks were constructed using network v. 10 (www.fluxus-engineering.com/sharenet.htm) and applying the median-joining and maximum parsimony options. Seven sublineages were found in the present study, and the diversity indices of each population and sublineage were calculated using DNASP v6.12.03. Two neutrality statistics, Tajima’s *D* and Fu’s *Fs* were also calculated with 1,000 permutations in ARLEQUIN v. 3.5.2.2. Analysis of molecular variance (AMOVA) was conducted in ARLEQUIN to partition the genetic variance within and among populations. Furthermore, four groups were defined according to the river system and Mountains system (HS1-4 and TH1-4 as Yellow River system in Northeastern QTP; JLJ1-6 as Yangtze River system in Northeastern QTP; LBW, LSH, LSM, LNS, YYJ, YYS, XA, LCB, LCC, and SXY as Yellow River system in Qinling Mountains; the other four populations as River systems in Southern Taihang Mountains) to estimate variance components.

### 2.3 Demographic history and gene flow

As seven sublineages of *T. robusta* were found in phylogenetic analyses. We used the *cytb* data to generate Bayesian skyline plots (BSP) to study the demographic history of each sublineage alone and the whole combined data set. The appropriate substitution model was determined as GTR with Moldetest v.3.7. We created individual input files for each sublineage and all combined with BEAUti available in the BEAST package. Analyses were run in BEAST v.2.6.7. We used a strict clock with the average evolution rate of 0.68%–0.86% per million years for Cobitidae estimated for *cytb* under the GTR model (Perdices et al., 2014). The program was run for 20 million generations sampling every 2,000 generations. The Bayesian Skyline plots were subsequently generated with TRACER v.1.7.1.

Pairwise migration rates between the seven sublineages were estimated using the *cytb* and a maximum likelihood coalescent approach implemented in MIGRATE-n v 3.6.4; we estimated Θ and *M* (immigration rate/mutation rate) based on *F*_ST_ values. We ran 10 short chains with a total of 10,000 genealogy samples and three long chains with 1,000,000 samples, following a burn-in of 10,000 samples; four independent runs were performed.

### 2.4 Divergence time estimation

The divergence time was estimated using a molecular clock approach as implemented in BEAST v2.6.7 with a relaxed clock model. We used a reduced dataset of *cytb*: specimens for which three to six sequences from each population were included. This method resulted in 106 terminals—98 representatives of the 28 populations of *T. robusta* and eight outgroup sequences (*T*. *siluroides* and *T*. *strauchii*) (Supplementary Table S2). As fossil evidence for *Triplophysa* was lacking, we used a conservative approach by employing two calibration points from previous studies (Feng et al., 2019; Wu et al., 2020). The most recent common ancestor (TMRCA) of *Triplophysa* dates from the early Miocene, corresponding to the early Miocene QTP uplift (Wu et al., 2020). The prior “speciation: Yule process” tree was used to construct the tree. We ran four independent runs for 20 million generations logging trees every 2000 generations. Convergence was checked with TRACER v. 1.7.1. The maximum credibility tree was created in TreeAnnotator v. 2.6.7 available in the BEAST package.

### 2.5 Reconstruction of ancestral areas

A biogeographic reconstruction of ancestral areas using BioGeoBEARS was performed in RASP 4.02. Ancestral distributions were reconstructed using 31 *cytb* sequences covering the range of seven sublineages of *T. robusta* (Supplementary Table S3). The 7,500 post-burn-in trees resulting from the BEAST analysis were integrated for inference. Five major areas for *T. robusta* according to our collection localities and the published literature (Wang et al., 2016; Li et al., 2017; Feng et al., 2019; Wu et al., 2020) are depicted as follows: (A) Upper Yellow River system in Northeastern QTP, (B) Jialing River system in Northeastern QTP, (C) Yellow River system in Qinling Mountains, (D) Yellow River system in Southern Taihang Mountains, and (E) Haihe River system in Southern Taihang Mountains. All six models of geographic range evolution were compared in a likelihood framework. The DIVALIKE + *j* was chosen by comparing Akaike’s information criterion and likelihood-ratio tests.

### 2.6 Species distribution and paleo-distribution modeling

We used species distribution modeling (SDM) to construct a model of current, Last Glacial Maximum (LGM, about 22,000 years ago), and Last Interglacial (LIG; ∼120,000–140,000 years before present) *T. robusta* distributions. The occurrence records for *T. robusta* were collected from all published literature about *T. robusta* (Li et al., 2017; Feng et al., 2019; Wu et al., 2020), and a total of 29 unique, geo-referenced and quality-checked occurrence records were finally retained in the present study (Supplementary Table S4).

We extracted current bioclimatic data from the WORLDCLIM dataset (http://www.worldclim.org/) at 30 arc-seconds resolution, LGM bioclimatic data from the Model for Interdisciplinary Research on Climate (MIROC) dataset at 2.5-min resolution (Hasumi and Emori, 2004), and LIG bioclimatic data from Otto-Bliesner et al. (2006) at 30 arc-seconds resolution. The ArcGIS 10.0 (ESRI: Redlands, CA) was used to extract climatic variable layers to include the distribution area (QTP and adjacent area, Qinling Mountains, and Taihang Mountains) of *T. robusta* to improve the predictive power of Maxent models (Anderson and Raza, 2010). All layers were clipped to an extent encompassing the known range of *T. robusta*, as well as adjacent, potentially accessible habitats (30–40° N and 98–116° E). Prior to constructing SDM, the ENMTools v. 1.3 was used to determine which bioclimatic variables were correlated, using R > 0.90 as a cutoff, and fifteen variables (bio1, bio2, bio5, bio6, bio8, bio9, bio10, bio11, bio12, bio13, bio14, bio16, bio17, bio18, and bio19) which were found to be correlated with at least one other variable were removed. The following four predictor variables were retained: bio3, 4, 7, and 15. Using the four Bioclim layers and spatially filtered occurrence data, We ran the Maxent v. 3.4.4 to construct a present-day SDM, and then projected it to the LIG and LGM climate conditions, and used the following settings for the Maxent model: hinge features only, regularization multiplier of 1,10,000 max number of background points, replicate the run type of 10 cross-validations (equivalent to 20% testing), logistic output, 500 maximum iterations, and 0.00001 convergence threshold, and a random seed. Furthermore, we optimized the regularization multiplier by generating models with values from 1 to 10 and chose the value with the best Akaike Information Criterion score. We ran jackknife tests to measure the importance of each bioclimatic variable. Models used 29 records for testing and the four BIOCLIM environmental layers to produce models for present and paleodistributions of *T. robusta*. The logistic output, which was a continuous probability of presence ranging from 0 to 1, was displayed in ARCGIS 10.0.

## 3 Results

### 3.1 Phylogeographic pattern of *T. robusta*

Both phylogenetic tree (ML and BI) and haplotype network showed a clear identification of seven sublineages found in *T. robusta* (Figure 1). There was no shared haplotype among the six sublineages (L1-L6) in different river systems and mountains, except for the sublineage L7 in upstream of Yellow River and Jialing River. Therefore, a distinct biogeographic structure with six particular geographical regions was found across the investigation area, two in Northeastern QTP (sublineage L5 and L6), three in Eastern Qinling Mountains (sublineage L1, L2, and L3), and one in South Taihang Mountains (sublineage L4). However, sublineage L7 had a wide distribution across the upstream of Yellow River and Jialing River. Low levels of diversity indices were found in each sublineage (π < 0.005, Supplementary Table S5) and in most populations (Table 1). Particularly, the very low genetic diversity (π < 0.001, Hd < 0.500) was found in populations YYJ and YYS, distributed in the Luohe River system. Tajima’s *D* and Fu’s *Fs* were not significant in any sublineages, except for sublineage L7 (*p* < 0.01), as well as for all combined populations (Supplementary Table S5). AMOVA revealed that only 39.37% of genetic variation was explained by intra-population variation, while 60.63% (*p* < 0.0000) explained variation among populations (Table 2). Furthermore, a high differentiation at the group level was found in the present study, and 56.25% (*p* < 0.0000) explained variation among groups (Table 2). All these above results clearly identified a distinct hierarchical phylogeographical structure for the *T. robusta* in the studied area.

**TABLE 2:** Analysis of molecular variance partitioning the genetic variance within and among populations, and molecular variance results comparing genetic variation among four groups (HS1-4 and TH1-4 as Yellow River system in Northeastern QTP; JLJ1-6 as Yangtze River system in Northeastern QTP; LBW, LSH, LSM, LNS, YYJ, YYS, XA, LCB, LCC, and SXY as Yellow River system in Qinling Mountains; other four populations as River systems in Southern Taihang Mountains).

Source of variation	Df	Sum of squares	Variance components	Percentage of variation with *p* value
Among populations	3	1285.646	7.38772 Va	60.63
Within populations	230	1103.277	4.79686 Vb	39.37
Total	233	2,388.923	12.18457	

Among four groups	3	1285.646	6.84779 Va	56.25
Among populations within groups	23	610.985	2.94756 Vb	24.21
Within populations	207	492.292	2.37822 Vc	19.54
Total	233	2388.923	12.17358	

### 3.2 Demographic history and gene flow

The BSP detected relatively stable population sizes throughout the last 0.22 Mya for most sublineages (L1-L6) (data were not shown). However, the population size of sublineage L7 appeared to have increased constantly since 0.18 Mya (Supplementary Figure S1). Furthermore, the overall population size of the species appeared to have rapidly increased constantly since 0.21 Mya (Supplementary Figure S2). This suggested that only sublineage L7 in Northeastern QTP and the combined populations had expanded historically, coinciding with the result of the neutrality test (Supplementary Table S5).

Estimates of gene flow calculated with MIGRATE-n indicated low to high levels of historical gene flow between sublineages (Supplementary Table S6). The effective number of migrants entering and leaving each sublineage per generation ranged from *M* = 0.29 (L5→L4) to 15.44 (L5→L7). The highest unidirectional estimates of gene flow were found towards the sublineage L7 (*M* = 37.41) and the lowest was in sublineage L4 (*M* = 2.04), while the highest gene flow out of a sublineage was from L5 (*M* = 18.14).

### 3.3 Divergence time estimation and ancestral range reconstruction

The time-calibrated molecular clock analyses dated the TMRCA of *T. robusta* and *T. siluroides* at approximately 8.51 Mya (95% highest posterior density HPD: 10.62–6.61 Mya) (Supplementary Figure S3). The divergence between sublineage L1 (Yihe River) and sublineage L2 (Luohe River) distributed in Eastern Qinling Mountains was at approximately 0.70 Mya, and both sublineages diverged from sublineages L5-7 in Northeastern QTP at approximately 2.17 Mya (HPD: 2.84–1.58 Mya). Sublineage L3 in Eastern Qinling Mountains split from L4 in South Taihang Mountains at approximately 0.61 Mya, and both sublineages diverged from L5-7 at approximately 1.00 Mya. Sublineage L5 upstream of the Yellow River diverged from L6 in Jialing River at approximately 0.65 Mya, and both diverged from sublineage L7 at approximately 0.87 Mya.

Ancestral range reconstruction showed that TMRCA of *T. robusta* was mainly distributed in the Yellow River basin (node 61, AC: 45.87%, Supplementary Figure S4). The Clade I most likely originated in the Eastern Qinling Mountains, while the Clade II originated in Northeastern QTP, indicating a vicariant event early in the history of *T. robusta*.

### 3.4 Species distribution modeling

**FIGURE 2:**
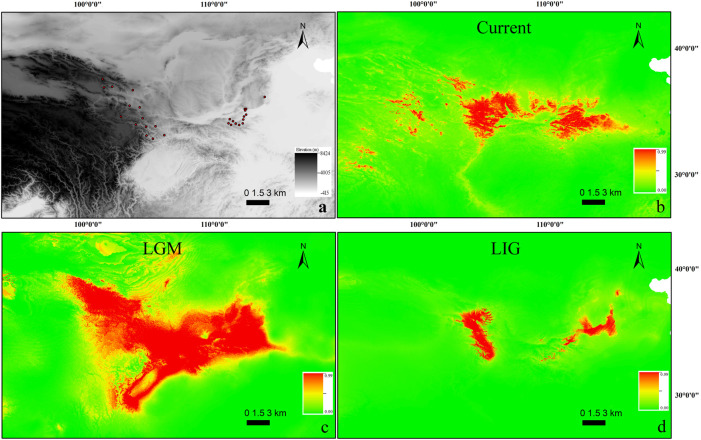
**(A)** Occurrence points used for building contemporary SDM for *T. robusta* (*n* = 29). The scale bar depicts altitude values. **(B–D)**: Species distribution modeling (SDM) for *T. robusta* during the current **(B)**, LGM **(C)**, and LIG **(D)** periods. Scale bars depict suitability values.

Maxent modeling accurately predicted a current range similar to that known for *T. robusta* in China with little variance (Figures 2A,B), with AUC values of 0.967 (SD = 0.020; training AUC range: 0.972–0.980, test AUC range: 0.925–0.990). The temperature seasonality (bio4; 49.9%), Precipitation Seasonality (bio15; 24.4%), were the largest contributors to the model contributing 74.3%, however, Temperature Annual Range (bio7) and bio4 were the highest permutation importance (31.8%, 27.1%, respectively) as supported by jackknifing.

When the model used current conditions to predict suitable habitat for *T. robusta* during LGM, the main areas of suitable habitat with high probability for this species were as follows: the whole Qinling Mountains, Northeastern QTP, and Southern Taihang Mountains (Figure 2C). However, during LIG, suitable habitats reflected that of LIG were very different from LGM and the present distribution, with a greater concentrtion of suitable habitats in the Qinling Mountains and Northeastern QTP (Figure 2D).

## 4 Discussion

As a typical montane species, *T. robusta* is mainly distributed in Northeastern QTP and Qinling Mountains, providing an interesting model to explore the impact of paleoclimate oscillation and geologic events on diversification patterns. It was not uncommon that species restricted to high elevation mountain habitats often show high levels of intra-species divergence and even unique patterns of genetic structure (Pauls et al., 2006; Hou et al., 2014; de Oliveira et al., 2021). We found seven distinct sublineages of *T. robusta* with no shared haplotype among different mountain rivers, even in two close mountain rivers (Yihe River and Luohe River) in Qinling Mountains. However, we found two shared haplotypes (h33 and h62, Figure 1) distributed upstream of the Yellow River and Jialing River, which are divided by watershed in Northeastern QTP. The present study showed a novel phylogeographic pattern and evolution history for *T. robusta* compared with previous studies on this species. The interspecies hybridization and introgressions in the *T. robusta* complex have distorted the molecular systematics and restricted speciation in the *T. robusta* complex (Feng et al., 2019). However, our study emphasized the historical gene flow among populations of *T. robusta*, resulting from the repetition of the glacial period during the Pleistocene, which played a crucial role in the phylogeographic pattern of *T. robusta*. Furthermore, our results showed that TMRCA of *T. robusta* differentiated into two clades (Figure 1) caused by a vicariant event early in the history of *T. robusta*, and Clade I most likely originated in the Eastern Qinling Mountains. While it was speculated that *T. robusta* in Qinling Mountains was mostly derived from the upstream of the Yellow River (Wu et al., 2020).

Our results revealed that *T. robusta* experienced Pleistocene rapid radiation. Divergence time among the seven sublineages was estimated at 2.17–0.61 Mya. Previous studies on plants and animals suggested mountain building as a predominant driver in triggering biological evolution (Hoorn et al., 2010; Smith et al., 2014; Simões et al., 2016), especially for the diversification of the species in QTP and Qinling Mountains (Che et al., 2010; Yan et al., 2010; Xu et al., 2018). However, there were few studies on the intra-species diversification between the two mountain lineages. In this study, we recovered the *T. robusta* in the investigated region of the present study with two main clades separated by the Qinling Mountains (Figure 1, Supplementary Figure S3). This mountain range extends more than 1,500 km from West to East in Central China with an average elevation between 1,500–3,000 m, representing an important geological and geographical boundary (Yan et al., 2010; Xu et al., 2018). The DIVALIKE + *j*-based analyses estimated allopatric speciation with the two sister clades inheriting each half of the ancestral range (Clade I in Eastern Qinling Mountains and Clade II in Northeastern QTP) (Supplementary Figure S4). The divergence time between clade I and clade II was estimated at approximately 2.17 Mya, relating to the further rapid uplift of the Qinling Mountains that occurred between 2.4 and 1.2 Mya (Yuan et al., 2012), and the interglacial period between 2.5 and 1.8 Mya (Xing, 1989; Jing and Liu, 1999). The retraction of cold-adapted species restricted to higher elevations during interglacial periods may have been another crucial element for producing a phylogeographic pattern for montane species (de Oliveira et al., 2021). The vicariance event likely had repeatedly happened during Pleistocene interglacials in Northeastern QTP and Qinling Mountains. Feng et al. (2019) mainly investigated the molecular systematics of the *T. robusta* complex distributed in Northeastern QTP. They speculated that there existed hybridization and introgressions between sympatric species according to the mitonuclear discordances, such as the hybridization and introgressions between *T. minxianensis* and *T. robusta*, and between *T. pappenheimi* and *T. siluroides.* Wu et al. (2020) studied the molecular phylogeny and divergence of *Triplophysa* species distributed in the Qinling Mountains and Southern Taihang Mountains. However, the phylogeography pattern of *T. robusta* is still unknown according to the two previous studies. Fortunately, they supply a comprehensive distribution for the *T. robusta* in China, making a good opportunity for us to investigate the phylogeography of *T. robusta* across Northeastern QTP and the Qinling Mountains*.* The SDM suggested increases in habitat suitability for *T. robusta* during LGM (Figure 2C), but contraction during LIG (Figure 2D), especially in Qinling Mountains. Unidirectional migration was found in *T. robusta*, with middle to high level of *M* (>1.00) from sublineages (L1-L4) in Eastern Qinling Mountains to that (L5 and L7) in Northeastern QTP (Supplementary Table S6). This scenario of historical gene flow from east to west supported the existence of historical corridors for the dispersion of high-elevation species during periods of climate cooling (Antonelli et al., 2010; de Oliveira et al., 2021). However, we found a low level of migration rate in the opposite direction (a West-East pattern), which was uncommon in freshwater species found in China during the same or much older period, such as the frogs, snails, and fish (Yan et al., 2013; Gu et al., 2019; Gu et al., 2022). The authors suggested the role of Pleistocene interglacials dispersal in influencing the species diversification.

In the present study, the divergence estimated for the seven sublineages showed rapid radiation during 0.87–0.61 Mya (Supplementary Figure S3), exactly coinciding with the Pleistocene interglacial stage (0.90–0.40 Mya) in China. It was reported that the main uplift events of the Qinling Mountains with an elevation of about 3,000 m (Zhu, 1989) had been completed before the Early Pleistocene (1.67–1.45 Mya). The orogenic belt of the Taihang Mountains, with an average elevation of 1,500–2000 m, rose sharply to approximately 1,400 m at the Miocene/Pliocene boundary, and reached their current elevation at the end of the Pliocene (Gong, 2010). Many speciation events and intraspecific differentiation might have been driven by the massive uplifting of the Taihang Mountains and Qinling Mountains from the late Miocene to the Pliocene. Species such as the freshwater *Gammarus* with low dispersal ability showed a congruent pattern with geographical vicariance caused by the uplift of the Lüliang and Taihang Mountains (Hou et al., 2014). The uplift of the Qinling Mountains during 23–2.6 Ma as a vicariance event had driven the speciation of *Sinothela* (Xu et al., 2018). However, the recent Mid-Pleistocene diversifications in different sublineages of *T. robusta* were far postdated to the uplift of the two mountains. Therefore, the retractions during inter-glacial periods found in the present study were more likely to play a key role in interrupting gene flow for cold-adapted species. It was likely a good candidate explanation of the Mid Pleistocene rapid diversification for this species.

Strangely, sublineage L7 in Northeastern QTP had a wide distribution across the upstream of Jialing River and Yellow River, in which both possessed their own sublineages (L5 and L6, respectively). The nested genetic structure of *T. robusta* in Northeastern QTP suggested an exchange between the Yellow River and the Jialing River. The high level of historical gene flow from Jialing River to Yellow River supported the existence of historical corridors for the dispersal of *T. robusta* (Supplementary Table S6) and coincided with the speculation that the upstream of Yellow River was once connected to the Jialing River. It was supposed that the continual tectonic activities, such as earthquakes over the past million years in the Northeastern QTP likely led to extensive gene flow (Feng et al., 2019). Furthermore, during glacial periods, ephemeral rivers and periglacial lakes could arise, likely to provide further opportunities for dispersal and interactions between populations of *T. robusta*. The elevation shift model stated that populations of cold-adapted species inhabiting high elevations expanded to lowland areas during periods of climate cooling, increasing spatial connectivity and gene flow (Galbreath et al., 2009; Newman and Austin, 2015; de Oliveira et al., 2021).

Previous studies on the *T. robusta* (Feng et al., 2019; Wu et al., 2020) have little practical and instructive value on the conservation strategies of wildly distributed species of *Triplophysa*. The present study had an important implication for the conservation of the cold-adapted species restricted to higher elevations. The diversification pattern found in the *T. robusta* can provide guidelines for effective conservation measures for *Triplophysa*, which might be negatively impacted by current climate change caused by humans. Montane endemic species were thought to be particularly vulnerable to the effects of rapid climate changes, as lots of montane habitats may be lost or fragmented due to climate warming (Moritz et al., 2008). Bonatelli et al. (2014) and Fernandes et al. (2014) believed that climate change in the next few decades might accelerate the declines and local extinctions of high elevation species due to the reduction of suitable habitats. The low genetic diversity found in most sublineages and populations of *T. robusta* indicated a serious challenge in adaptive ability (O’Brien, 1994; Bazin et al., 2006). Our results suggested that the temperature was one of the most important environmental factors influencing *T. robusta* distribution range, which experienced a considerable retraction as historical climatic fluctuations in Northeastern QTP and Qinling Mountains in China. In this context, the studied species *T. robusta*, as well as other *Triplophysa* distributed in the QTP and adjacent regions would be negatively impacted by climate warming. A burning question in future conservation projection on cold-adapted fish species endemic to higher elevations is how to tackle global warming for them. Furthermore, mid to low levels of genetic diversities were found for all populations except for JYT (Table 1). While *T. robusta* is classed as Least Concern (LC) in China, the current conservation strategy is carried out under the assumption of only one species and one gene pool. Our results demonstrated that different protection strategies for *T. robusta* should be conducted in different mountains and river systems: two protection units for *T. robusta* in Northeastern QTP, one upstream of the Yellow River basin, and the other one upstream of the Jialing River; three protection units in Qinling Mountains, one in Yihe River, one in Luohe River and another in Dongjian River; and only one protection unit in the Southern Taihang Mountains. Especially for two populations (YYJ and YYS) in a couple of mountain streams in Luohe River, with very low nucleotide diversity and haplotype diversity, forbidden fishing zone and habitat environment restoration should be implemented to strengthen protection for them.

## Data Availability

The datasets presented in this study can be found in online repositories. The names of the repository/repositories and accession numbers can be found in the article/Supplementary Material.
