# Supplementary material for: Pleistocene climate and geomorphology drive the evolution and phylogeographic pattern of Triplophysa robusta (Kessler, 1876)
Source: Front Genet. 2022 Sep 12;13:955382. doi: 10.3389/fgene.2022.955382 (PMC9510703; doi:10.3389/fgene.2022.955382)
Supplement: Supplementary file 1 [file DataSheet2.docx]

Table S5 Diversity indices and neutrality test for seven sublineages of *T. robusta* in China based on cytb

| Sublieages | N | S | H | Hd | π | P | Neutrality test | |
| --- | --- | --- | --- | --- | --- | --- | --- | --- |
|  |  |  |  |  |  |  | Tajima’s *D* | Fu’s *Fs* |
| L1 | 19 | 20 | 13 | 0.942 | 0.00295 | 9 | -1.64545 | -6.607 |
| L2 | 74 | 39 | 27 | 0.924 | 0.00390 | 24 | -1.52816 | -11.284 |
| L3 | 6 | 3 | 3 | 0.800 | 0.00144 | 2 | 0.69900 | 0.276 |
| L4 | 14 | 7 | 8 | 0.912 | 0.00199 | 5 | 0.01278 | -2.973 |
| L5 | 5 | 6 | 5 | 1.000 | 0.00216 | 0 | -1.14554 | -2.680 |
| L6 | 16 | 9 | 9 | 0.883 | 0.00181 | 3 | -0.95323 | -4.166 |
| L7 | 100 | 67 | 55 | 0.968 | 0.00391 | 34 | -2.16619** | -33.181** |
| Total | 234 | 153 | 121 | 0.986 | 0.01846 | 108 | -0.64631 | -33.271** |

N indicates the sequences number, S indicates the number of polymorphic sites, H indicates the number of haplotypes, Hd indicates haplotype diversity, π indicates nucleotide diversity, P indicates parsimony informative sites, **statistical significance, *P* < 0.05.

Table S6 Immigration rates (M) into each of the 7 *T. robusta* sublineages from every other sublineage as estimated by MIGRATE-n

| pop  *i* |  | L1 | L2 | L3 | L4 | L5 | L6 | L7 |  |
| --- | --- | --- | --- | --- | --- | --- | --- | --- | --- |
|  | Θ*_i_* | L1→*i* | L2→*i* | L3→*i* | L4→*i* | L5→*i* | L6→*i* | L7→*i* | Total→*i* |
| L1 | 0.0086 |  | 0.78 | 0.91 | 0.72 | 0.93 | 0.89 | 0.70 | 4.92 |
| L2 | 0.0105 | 0.36 |  | 0.44 | 0.42 | 0.40 | 0.39 | 0.56 | 2.58 |
| L3 | 0.0013 | 0.46 | 0.46 |  | 0.47 | 0.46 | 0.47 | 0.65 | 2.97 |
| L4 | 0.0012 | 0.31 | 0.29 | 0.32 |  | 0.29 | 0.38 | 0.45 | 2.04 |
| L5 | 0.0067 | 1.44 | 1.27 | 1.94 | 1.41 |  | 1.73 | 2.66 | 10.45 |
| L6 | 0.0026 | 0.56 | 0.58 | 0.70 | 0.63 | 0.63 |  | 0.62 | 3.72 |
| L7 | 0.0442 | 1.52 | 1.40 | 7.56 | 10.50 | 15.44 | 0.99 |  | 37.41 |
| Total |  | 4.65 | 4.77 | 11.87 | 14.16 | 18.14 | 4.85 | 5.64 |  |

*i* means this sublineage, L1→*i*, means unidirectional migrate rate between sublineage L1 and other sublineages, and the Θ*_i_* means the parameter of migrate rate in each generation;
