# Supplementary material for: Pleistocene climate and geomorphology drive the evolution and phylogeographic pattern of Triplophysa robusta (Kessler, 1876)
Source: Front Genet. 2022 Sep 12;13:955382. doi: 10.3389/fgene.2022.955382 (PMC9510703; doi:10.3389/fgene.2022.955382)
Supplement: Supplementary file 2 [file DataSheet1.docx]

**Table S1.** Detailed information for specimens included in this study, NA means absent in present study. Sequences for the present article are ON630917-ON630958. Accession numbers with “MG” prefix are sequences from Feng et al. (2019), and “MN” prefix are sequences from Wu et al., (2020), retrieved from the GenBank.

| **Species** | **Location** | **ID** | **River system** | ***16S*** | ***COI*** | ***Cyt b*** | ***RH1*** |
| --- | --- | --- | --- | --- | --- | --- | --- |
| **Ingroup** |  |  |  |  |  |  |  |
| *T. robusta* | HS1 | F_Y322 | Yellow River | MG697991 | NA | MG697367 | MG697780 |
| *T. robusta* | HS1 | F_Y323 | Yellow River | MG697992 | NA | MG697368 | MG697781 |
| *T. robusta* | HS1 | F_Y325 | Yellow River | MG697993 | NA | MG697369 | MG697785 |
| *T. robusta* | HS1 | F_Y326 | Yellow River | MG697994 | NA | MG697370 | MG697786 |
| *T. robusta* | HS1 | F_Y327 | Yellow River | MG697995 | NA | MG697371 | MG697787 |
| *T. robusta* | HS1 | F_Y328 | Yellow River | MG697996 | NA | MG697372 | MG697788 |
| *T. robusta* | HS1 | F_Y329 | Yellow River | MG697997 | NA | MG697373 | MG697789 |
| *T. robusta* | HS1 | F_Y330 | Yellow River | MG697998 | NA | MG697374 | MG697790 |
| *T. robusta* | HS1 | F_Y331 | Yellow River | MG697999 | NA | MG697375 | MG697794 |
| *T. robusta* | HS1 | F_Y332 | Yellow River | MG698000 | NA | MG697376 | MG697795 |
| *T. robusta* | HS1 | F_Y333 | Yellow River | MG698001 | NA | MG697377 | MG697796 |
| *T. robusta* | HS1 | F_Y334 | Yellow River | MG698002 | NA | MG697378 | MG697797 |
| *T. robusta* | HS1 | F_Y749 | Yellow River | MG698008 | NA | MG697384 | MG697885 |
| *T. robusta* | HS2 | F_Y494 | Yellow River | MG698010 | NA | MG697386 | MG697877 |
| *T. robusta* | HS2 | F_Y3875 | Yellow River | MG698011 | NA | MG697387 | MG697846 |
| *T. robusta* | HS2 | F_Y3876 | Yellow River | MG698012 | NA | MG697388 | MG697847 |
| *T. robusta* | HS2 | F_Y3877 | Yellow River | MG698013 | NA | MG697389 | MG697848 |
| *T. robusta* | HS2 | F_Y3878 | Yellow River | MG698014 | NA | MG697390 | MG697849 |
| *T. robusta* | HS2 | F_Y3879 | Yellow River | MG698015 | NA | MG697391 | MG697850 |
| *T. robusta* | HS2 | F_Y3880 | Yellow River | MG698016 | NA | MG697392 | MG697851 |
| *T. robusta* | HS2 | F_Y3881 | Yellow River | MG698017 | NA | MG697393 | MG697852 |
| *T. robusta* | HS3 | F_Y3852 | Yellow River | MG698018 | NA | MG697394 | MG697843 |
| *T. robusta* | HS3 | F_Y3853 | Yellow River | MG698019 | NA | MG697395 | MG697844 |
| *T. robusta* | HS3 | F_Y3854 | Yellow River | MG698020 | NA | MG697396 | MG697845 |
| *T. robusta* | HS4 | F_Y3668 | Yellow River | MG698021 | NA | MG697397 | MG697833 |
| *T. robusta* | HS4 | F_Y3669 | Yellow River | MG698022 | NA | MG697398 | MG697834 |
| *T. robusta* | HS4 | F_Y3670 | Yellow River | MG698023 | NA | MG697399 | MG697835 |
| *T. robusta* | HS4 | F_Y3671 | Yellow River | MG698024 | NA | MG697400 | MG697836 |
| *T. robusta* | HS4 | F_Y3672 | Yellow River | MG698025 | NA | MG697401 | MG697837 |
| *T. robusta* | HS4 | F_Y3673 | Yellow River | MG698026 | NA | MG697402 | MG697838 |
| *T. robusta* | HS4 | F_Y3674 | Yellow River | MG698027 | NA | MG697403 | MG697839 |
| *T. robusta* | TH1 | F_Y2517 | Yellow River | MG698028 | NA | MG697404 | MG697659 |
| *T. robusta* | TH1 | F_Y2579 | Yellow River | MG698029 | NA | MG697405 | MG697681 |
| *T. robusta* | TH1 | F_Y2580 | Yellow River | MG698030 | NA | MG697406 | MG697682 |
| *T. robusta* | TH1 | F_Y2581 | Yellow River | MG698031 | NA | MG697407 | MG697683 |
| *T. robusta* | TH1 | F_Y3577 | Yellow River | MG698032 | NA | MG697408 | MG697827 |
| *T. robusta* | TH1 | F_Y3578 | Yellow River | MG698033 | NA | KX373850 | MG697828 |
| *T. robusta* | TH1 | F_Y3579 | Yellow River | MG698034 | NA | MG697409 | MG697829 |
| *T. robusta* | TH1 | F_Y3620 | Yellow River | MG698035 | NA | MG697410 | MG697830 |
| ***Species*** | **Location** | **ID** | **River system** | ***16S*** | ***COI*** | ***Cyt b*** | ***RH1*** |
| *T. robusta* | TH1 | F_Y3621 | Yellow River | MG698036 | NA | MG697411 | MG697831 |
| *T. robusta* | TH1 | F_Y3622 | Yellow River | MG698037 | NA | MG697412 | MG697832 |
| *T. robusta* | TH2 | F_Y2644 | Yellow River | MG698038 | NA | MG697413 | MG697685 |
| *T. robusta* | TH2 | F_Y2645 | Yellow River | MG698039 | NA | MG697414 | MG697686 |
| *T. robusta* | TH2 | F_Y2646 | Yellow River | MG698040 | NA | MG697415 | MG697687 |
| *T. robusta* | TH2 | F_Y2647 | Yellow River | MG698041 | NA | MG697416 | MG697688 |
| *T. robusta* | TH2 | F_Y2648 | Yellow River | MG698042 | NA | MG697417 | MG697689 |
| *T. robusta* | TH2 | F_Y2649 | Yellow River | MG698043 | NA | MG697418 | MG697690 |
| *T. robusta* | TH2 | F_Y2650 | Yellow River | MG698044 | NA | MG697419 | MG697691 |
| *T. robusta* | TH2 | F_Y2651 | Yellow River | MG698045 | NA | MG697420 | MG697692 |
| *T. robusta* | TH2 | F_Y2652 | Yellow River | MG698046 | NA | MG697421 | MG697693 |
| *T. robusta* | TH2 | F_Y2653 | Yellow River | MG698047 | NA | MG697422 | MG697694 |
| *T. robusta* | TH2 | F_Y2654 | Yellow River | MG698048 | NA | MG697423 | MG697695 |
| *T. robusta* | TH2 | F_Y2655 | Yellow River | MG698049 | NA | MG697424 | MG697696 |
| *T. robusta* | TH2 | F_Y2656 | Yellow River | MG698050 | NA | MG697425 | MG697697 |
| *T. robusta* | TH2 | F_Y2675 | Yellow River | MG698051 | NA | MG697426 | MG697698 |
| *T. robusta* | TH2 | F_Y2676 | Yellow River | MG698052 | NA | MG697427 | MG697699 |
| *T. robusta* | TH2 | F_Y2677 | Yellow River | MG698053 | NA | MG697428 | MG697700 |
| *T. robusta* | TH3 | F_Y2905 | Yellow River | MG698054 | NA | MG697429 | MG697717 |
| *T. robusta* | TH3 | F_Y2906 | Yellow River | MG698055 | NA | MG697430 | MG697718 |
| *T. robusta* | TH3 | F_Y2907 | Yellow River | MG698056 | NA | MG697431 | MG697719 |
| *T. robusta* | TH3 | F_Y3048 | Yellow River | MG698066 | NA | MG697441 | MG697745 |
| *T. robusta* | TH3 | F_Y3050 | Yellow River | MG698068 | NA | MG697443 | MG697747 |
| *T. robusta* | TH3 | F_Y3051 | Yellow River | MG698069 | NA | MG697444 | MG697748 |
| *T. robusta* | TH3 | F_Y3052 | Yellow River | MG698070 | NA | MG697445 | MG697749 |
| *T. robusta* | TH4 | F_Y2518 | Yellow River | MG698083 | NA | MG697457 | MG697660 |
| *T. robusta* | JLJ1 | F_J2519 | Jialing River | MG698153 | NA | MG697525 | MG697661 |
| *T. robusta* | JLJ1 | F_J2520 | Jialing River | MG698154 | NA | MG697526 | MG697662 |
| *T. robusta* | JLJ1 | F_J2521 | Jialing River | MG698155 | NA | MG697527 | MG697663 |
| *T. robusta* | JLJ1 | F_J2522 | Jialing River | MG698156 | NA | MG697528 | MG697664 |
| *T. robusta* | JLJ1 | F_J2523 | Jialing River | MG698157 | NA | MG697529 | MG697665 |
| *T. robusta* | JLJ1 | F_J2524 | Jialing River | MG698158 | NA | MG697530 | MG697666 |
| *T. robusta* | JLJ1 | F_J2525 | Jialing River | MG698159 | NA | MG697531 | MG697667 |
| *T. robusta* | JLJ1 | F_J2526 | Jialing River | MG698160 | NA | MG697532 | MG697668 |
| *T. robusta* | JLJ1 | F_J2527 | Jialing River | MG698161 | NA | MG697533 | MG697669 |
| *T. robusta* | JLJ1 | F_J2528 | Jialing River | MG698162 | NA | MG697534 | MG697670 |
| *T. robusta* | JLJ1 | F_J2529 | Jialing River | MG698163 | NA | MG697535 | MG697671 |
| *T. robusta* | JLJ1 | F_J2540 | Jialing River | MG698164 | NA | MG697536 | MG697675 |
| *T. robusta* | JLJ1 | F_J2541 | Jialing River | MG698165 | NA | MG697537 | MG697676 |
| *T. robusta* | JLJ1 | F_J2546 | Jialing River | MG698166 | NA | MG697538 | MG697677 |
| *T. robusta* | JLJ1 | F_J2547 | Jialing River | MG698167 | NA | MG697539 | MG697678 |
| *T. robusta* | JLJ1 | F_J2554 | Jialing River | MG698168 | NA | MG697540 | MG697679 |
| *T. robusta* | JLJ1 | F_J2556 | Jialing River | MG698169 | NA | MG697541 | MG697680 |
| **Species** | **Location** | **ID** | **River system** | ***16S*** | ***COI*** | ***Cyt b*** | ***RH1*** |
| *T. robusta* | JLJ2 | F_J2530 | Jialing River | MG698170 | NA | MG697542 | MG697672 |
| *T. robusta* | JLJ2 | F_J2531 | Jialing River | MG698171 | NA | MG697543 | MG697673 |
| *T. robusta* | JLJ2 | F_J2532 | Jialing River | MG698172 | NA | MG697544 | MG697674 |
| *T. robusta* | JLJ2 | F_J3058 | Jialing River | MG698173 | NA | MG697545 | MG697750 |
| *T. robusta* | JLJ2 | F_J3060 | Jialing River | MG698174 | NA | MG697546 | MG697751 |
| *T. robusta* | JLJ3 | F_J3362 | Jialing River | MG698175 | NA | MG697547 | MG697802 |
| *T. robusta* | JLJ3 | F_J3363 | Jialing River | MG698176 | NA | MG697548 | MG697803 |
| *T. robusta* | JLJ3 | F_J3364 | Jialing River | MG698177 | NA | MG697549 | MG697804 |
| *T. robusta* | JLJ4 | F_J3244 | Jialing River | MG698178 | NA | MG697550 | MG697782 |
| *T. robusta* | JLJ4 | F_J3245 | Jialing River | MG698179 | NA | MG697551 | MG697783 |
| *T. robusta* | JLJ4 | F_J3246 | Jialing River | MG698180 | NA | MG697552 | MG697784 |
| *T. robusta* | JLJ5 | F_J3172 | Jialing River | MG698181 | NA | MG697553 | MG697756 |
| *T. robusta* | JLJ5 | F_J3173 | Jialing River | MG698182 | NA | MG697554 | MG697757 |
| *T. robusta* | JLJ5 | F_J3174 | Jialing River | MG698183 | NA | MG697555 | MG697758 |
| *T. robusta* | JLJ5 | F_J3175 | Jialing River | MG698184 | NA | MG697556 | MG697759 |
| *T. robusta* | JLJ5 | F_J3176 | Jialing River | MG698185 | NA | MG697557 | MG697760 |
| *T. robusta* | JLJ5 | F_J3177 | Jialing River | MG698186 | NA | MG697558 | MG697761 |
| *T. robusta* | JLJ5 | F_J3178 | Jialing River | MG698187 | NA | MG697559 | MG697762 |
| *T. robusta* | JLJ5 | F_J3179 | Jialing River | MG698188 | NA | MG697560 | MG697763 |
| *T. robusta* | JLJ5 | F_J3180 | Jialing River | MG698189 | NA | MG697561 | MG697764 |
| *T. robusta* | JLJ5 | F_J3181 | Jialing River | MG698190 | NA | MG697562 | MG697765 |
| *T. robusta* | JLJ5 | F_J3182 | Jialing River | MG698191 | NA | MG697563 | MG697766 |
| *T. robusta* | JLJ5 | F_J3183 | Jialing River | MG698192 | NA | MG697564 | MG697767 |
| *T. robusta* | JLJ5 | F_J3184 | Jialing River | MG698193 | NA | MG697565 | MG697768 |
| *T. robusta* | JLJ5 | F_J3185 | Jialing River | MG698194 | NA | MG697566 | MG697769 |
| *T. robusta* | JLJ5 | F_J3186 | Jialing River | MG698195 | NA | MG697567 | MG697770 |
| *T. robusta* | JLJ5 | F_J3187 | Jialing River | MG698196 | NA | MG697568 | MG697771 |
| *T. robusta* | JLJ5 | F_J3188 | Jialing River | MG698197 | NA | MG697569 | MG697772 |
| *T. robusta* | JLJ5 | F_J3189 | Jialing River | MG698198 | NA | MG697570 | MG697773 |
| *T. robusta* | JLJ5 | F_J3190 | Jialing River | MG698199 | NA | MG697571 | MG697774 |
| *T. robusta* | JLJ5 | F_J3191 | Jialing River | MG698200 | NA | MG697572 | MG697775 |
| *T. robusta* | JLJ5 | F_J3192 | Jialing River | MG698201 | NA | MG697573 | MG697776 |
| *T. robusta* | JLJ5 | F_J3193 | Jialing River | MG698202 | NA | MG697574 | MG697777 |
| *T. robusta* | JLJ5 | F_J3194 | Jialing River | MG698203 | NA | MG697575 | MG697778 |
| *T. robusta* | JLJ6 | F_J3304 | Jialing River | MG698204 | NA | MG697576 | MG697791 |
| *T. robusta* | JLJ6 | F_J3305 | Jialing River | MG698205 | NA | MG697577 | MG697792 |
| *T. robusta* | JLJ6 | F_J3306 | Jialing River | MG698206 | NA | MG697578 | MG697793 |
| *T. robusta* | JLJ6 | F_J3343 | Jialing River | MG698207 | NA | MG697579 | MG697798 |
| *T. robusta* | JLJ6 | F_J3344 | Jialing River | MG698208 | NA | MG697580 | MG697799 |
| *T. robusta* | JLJ6 | F_J3345 | Jialing River | MG698209 | NA | MG697581 | MG697800 |
| *T. robusta* | LZL | LZL1 | Haihe River | NA | MN896118 | MN896385 | ON630917 |
| *T. robusta* | LZL | LZL2 | Haihe River | NA | MN896119 | MN896386 | ON630918 |
| *T. robusta* | LZL | LZL3 | Haihe River | NA | MN896120 | MN896387 | ON630919 |
| **Species** | **Location** | **ID** | Haihe River | ***16S*** | ***COI*** | ***Cyt b*** | ***RH1*** |
| *T. robusta* | LZL | LZL4 | Haihe River | NA | MN896121 | MN896388 | NA |
| *T. robusta* | LZL | LZL5 | Haihe River | NA | MN896122 | MN896389 | NA |
| *T. robusta* | LZL | LZL6 | Haihe River | NA | MN896123 | MN896390 | NA |
| *T. robusta* | LZL | LZL7 | Haihe River | NA | MN896124 | MN896391 | NA |
| *T. robusta* | LZL | LZL8 | Haihe River | NA | MN896125 | MN896392 | NA |
| *T. robusta* | LZL | LZL9 | Haihe River | NA | MN896126 | MN896393 | NA |
| *T. robusta* | LBW | LBW1 | Yellow River | NA | MN896127 | MN896394 | ON630920 |
| *T. robusta* | LBW | LBW2 | Yellow River | NA | MN896128 | MN896395 | ON630921 |
| *T. robusta* | LBW | LBW3 | Yellow River | NA | MN896129 | MN896396 | ON630922 |
| *T. robusta* | LBW | LBW4 | Yellow River | NA | MN896130 | MN896397 | NA |
| *T. robusta* | LBW | LBW5 | Yellow River | NA | MN896131 | MN896398 | NA |
| *T. robusta* | LBW | LBW6 | Yellow River | NA | MN896132 | MN896399 | NA |
| *T. robusta* | LCB | LCB1 | Yihe River | NA | MN896133 | MN896400 | ON630923 |
| *T. robusta* | LCB | LCB2 | Yihe River | NA | MN896134 | MN896401 | ON630924 |
| *T. robusta* | LCB | LCB3 | Yihe River | NA | MN896135 | MN896402 | ON630925 |
| *T. robusta* | LCB | LCB4 | Yihe River | NA | MN896136 | MN896403 | NA |
| *T. robusta* | LCC | LCC1 | Yihe River | NA | MN896137 | MN896404 | ON630926 |
| *T. robusta* | LCC | LCC2 | Yihe River | NA | MN896138 | MN896405 | ON630927 |
| *T. robusta* | LCC | LCC3 | Yihe River | NA | MN896139 | MN896406 | ON630928 |
| *T. robusta* | LCC | LCC4 | Yihe River | NA | MN896140 | MN896407 | NA |
| *T. robusta* | LCC | LCC5 | Yihe River | NA | MN896141 | MN896408 | NA |
| *T. robusta* | LCC | LCC6 | Yihe River | NA | MN896142 | MN896409 | NA |
| *T. robusta* | LCC | LCC7 | Yihe River | NA | MN896143 | MN896410 | NA |
| *T. robusta* | LCC | LCC8 | Yihe River | NA | MN896144 | MN896411 | NA |
| *T. robusta* | LCC | LCC9 | Yihe River | NA | MN896145 | MN896412 | NA |
| *T. robusta* | SXY | SXY1 | Yihe River | NA | MN896146 | MN896413 | ON630929 |
| *T. robusta* | SXY | SXY2 | Yihe River | NA | MN896147 | MN896414 | ON630930 |
| *T. robusta* | SXY | SXY3 | Yihe River | NA | MN896148 | MN896415 | ON630931 |
| *T. robusta* | SXY | SXY4 | Yihe River | NA | MN896149 | MN896416 | NA |
| *T. robusta* | SXY | SXY5 | Yihe River | NA | MN896150 | MN896417 | NA |
| *T. robusta* | SXY | SXY6 | Yihe River | NA | MN896151 | MN896418 | NA |
| *T. robusta* | LNS | LNS1 | Luohe River | NA | MN896152 | MN896419 | ON630932 |
| *T. robusta* | LNS | LNS2 | Luohe River | NA | MN896153 | MN896420 | ON630933 |
| *T. robusta* | LNS | LNS3 | Luohe River | NA | MN896154 | MN896421 | ON630934 |
| *T. robusta* | LNS | LNS4 | Luohe River | NA | MN896155 | MN896422 | NA |
| *T. robusta* | LNS | LNS5 | Luohe River | NA | MN896156 | MN896423 | NA |
| *T. robusta* | LNS | LNS6 | Luohe River | NA | MN896157 | MN896424 | NA |
| *T. robusta* | LNS | LNS7 | Luohe River | NA | MN896158 | MN896425 | NA |
| *T. robusta* | LNS | LNS8 | Luohe River | NA | MN896159 | MN896426 | NA |
| *T. robusta* | LNS | LNS9 | Luohe River | NA | MN896160 | MN896427 | NA |
| *T. robusta* | LSH | LSH1 | Luohe River | NA | MN896161 | MN896428 | ON630935 |
| *T. robusta* | LSH | LSH2 | Luohe River | NA | MN896162 | MN896429 | ON630936 |
| *T. robusta* | LSH | LSH3 | Luohe River | NA | MN896163 | MN896430 | ON630937 |
| **Species** | **Location** | **ID** | **River system** | ***16S*** | ***COI*** | ***Cyt b*** | ***RH1*** |
| *T. robusta* | LSM | LSM1 | Luohe River | NA | MN896164 | MN896431 | ON630938 |
| *T. robusta* | LSM | LSM2 | Luohe River | NA | MN896165 | MN896432 | ON630939 |
| *T. robusta* | LSM | LSM3 | Luohe River | NA | MN896166 | MN896433 | ON630940 |
| *T. robusta* | LSM | LSM4 | Luohe River | NA | MN896167 | MN896434 | NA |
| *T. robusta* | LSM | LSM5 | Luohe River | NA | MN896168 | MN896435 | NA |
| *T. robusta* | LSM | LSM6 | Luohe River | NA | MN896169 | MN896436 | NA |
| *T. robusta* | LSM | LSM7 | Luohe River | NA | MN896170 | MN896437 | NA |
| *T. robusta* | LSM | LSM8 | Luohe River | NA | MN896171 | MN896438 | NA |
| *T. robusta* | LSM | LSM9 | Luohe River | NA | MN896172 | MN896439 | NA |
| *T. robusta* | XA | XA1 | Luohe River | NA | MN896173 | MN896440 | ON630941 |
| *T. robusta* | XA | XA2 | Luohe River | NA | MN896174 | MN896441 | ON630942 |
| *T. robusta* | XA | XA3 | Luohe River | NA | MN896175 | MN896442 | ON630943 |
| *T. robusta* | XA | XA4 | Luohe River | NA | MN896176 | MN896443 | NA |
| *T. robusta* | XA | XA5 | Luohe River | NA | MN896177 | MN896444 | NA |
| *T. robusta* | XA | XA6 | Luohe River | NA | MN896178 | MN896445 | NA |
| *T. robusta* | XA | XA7 | Luohe River | NA | MN896179 | MN896446 | NA |
| *T. robusta* | XA | XA8 | Luohe River | NA | MN896180 | MN896447 | NA |
| *T. robusta* | XA | XA9 | Luohe River | NA | MN896181 | MN896448 | NA |
| *T. robusta* | XA | XA10 | Luohe River | NA | MN896182 | MN896449 | NA |
| *T. robusta* | XA | XA11 | Luohe River | NA | MN896183 | MN896450 | NA |
| *T. robusta* | XA | XA12 | Luohe River | NA | MN896184 | MN896451 | NA |
| *T. robusta* | XA | XA13 | Luohe River | NA | MN896185 | MN896452 | NA |
| *T. robusta* | XA | XA14 | Luohe River | NA | MN896186 | MN896453 | NA |
| *T. robusta* | XA | XA15 | Luohe River | NA | MN896187 | MN896454 | NA |
| *T. robusta* | XA | XA16 | Luohe River | NA | MN896188 | MN896455 | NA |
| *T. robusta* | YYJ | YYJ1 | Luohe River | NA | MN896189 | MN896456 | ON630944 |
| *T. robusta* | YYJ | YYJ2 | Luohe River | NA | MN896190 | MN896457 | ON630945 |
| *T. robusta* | YYJ | YYJ3 | Luohe River | NA | MN896191 | MN896458 | ON630946 |
| *T. robusta* | YYJ | YYJ4 | Luohe River | NA | MN896192 | MN896459 | NA |
| *T. robusta* | YYJ | YYJ5 | Luohe River | NA | MN896193 | MN896460 | NA |
| *T. robusta* | YYS | YYS1 | Luohe River | NA | MN896194 | MN896461 | ON630947 |
| *T. robusta* | YYS | YYS2 | Luohe River | NA | MN896195 | MN896462 | ON630948 |
| *T. robusta* | YYS | YYS3 | Luohe River | NA | MN896196 | MN896463 | ON630949 |
| *T. robusta* | YYS | YYS4 | Luohe River | NA | MN896197 | MN896464 | NA |
| *T. robusta* | YYS | YYS5 | Luohe River | NA | MN896198 | MN896465 | NA |
| *T. robusta* | YYS | YYS6 | Luohe River | NA | MN896199 | MN896466 | NA |
| *T. robusta* | YYS | YYS7 | Luohe River | NA | MN896200 | MN896467 | NA |
| *T. robusta* | YYS | YYS8 | Luohe River | NA | MN896201 | MN896468 | NA |
| *T. robusta* | YYS | YYS9 | Luohe River | NA | MN896202 | MN896469 | NA |
| *T. robusta* | JYD | JYD1 | Yellow River | NA | MN896203 | MN896470 | ON630950 |
| *T. robusta* | JYD | JYD10 | Yellow River | NA | MN896204 | MN896471 | NA |
| *T. robusta* | JYD | JYD2 | Yellow River | NA | MN896205 | MN896472 | ON630951 |
| *T. robusta* | JYD | JYD3 | Yellow River | NA | MN896206 | MN896473 | ON630952 |
| **Species** | **Location** | **ID** | **River system** | ***16S*** | ***COI*** | ***Cyt b*** | ***RH1*** |
| *T. robusta* | JYD | JYD4 | Yellow River | NA | MN896207 | MN896474 | NA |
| *T. robusta* | JYD | JYD5 | Yellow River | NA | MN896208 | MN896475 | NA |
| *T. robusta* | JYD | JYD6 | Yellow River | NA | MN896209 | MN896476 | NA |
| *T. robusta* | JYD | JYD8 | Yellow River | NA | MN896211 | MN896478 | NA |
| *T. robusta* | JYD | JYD9 | Yellow River | NA | MN896212 | MN896479 | NA |
| *T. robusta* | JYD | JYD11 | Yellow River | NA | MN896222 | MN896489 | NA |
| *T. robusta* | JYD | JYD12 | Yellow River | NA | MN896223 | MN896490 | NA |
| *T. robusta* | JYF | JYF1 | Yellow River | NA | MN896213 | MN896480 | ON630953 |
| *T. robusta* | JYF | JYF2 | Yellow River | NA | MN896214 | MN896481 | ON630954 |
| *T. robusta* | JYF | JYF3 | Yellow River | NA | MN896215 | MN896482 | ON630955 |
| *T. robusta* | JYF | JYF5 | Yellow River | NA | MN896217 | MN896484 | NA |
| *T. robusta* | JYF | JYF6 | Yellow River | NA | MN896218 | MN896485 | NA |
| *T. robusta* | JYF | JYF7 | Yellow River | NA | MN896219 | MN896486 | NA |
| *T. robusta* | JYF | JYF9 | Yellow River | NA | MN896221 | MN896488 | NA |
| *T. robusta* | JYT | JYT1 | Yellow River | NA | MN896224 | MN896491 | ON630956 |
| *T. robusta* | JYT | JYT2 | Yellow River | NA | MN896225 | MN896492 | ON630957 |
| *T. robusta* | JYT | JYT3 | Yellow River | NA | MN896226 | MN896493 | ON630958 |
| *T. robusta* | JYT | JYT4 | Yellow River | NA | MN896227 | MN896494 | NA |
| *T. robusta* | JYT | JYT5 | Yellow River | NA | MN896228 | MN896495 | NA |
| *T. robusta* | JYT | JYT6 | Yellow River | NA | MN896229 | MN896496 | NA |
| *T. robusta* | JYT | JYT7 | Yellow River | NA | MN896230 | NA | NA |
| *T. robusta* | JYT | JYT8 | Yellow River | NA | MN896231 | NA | NA |
| *T. robusta* | JYT | JYT9 | Yellow River | NA | MN896232 | MN896497 | NA |
| *T. robusta* | JYT | JYT10 | Yellow River | NA | MN896216 | MN896483 | NA |
| *T. robusta* | JYT | JYT11 | Yellow River | NA | MN896220 | MN896487 | NA |
| *T. robusta* | JYT | JYT12 | Yellow River | NA | MN896210 | MN896477 | NA |
| **Outgroup** |  |  |  |  |  |  |  |
| *T. siluroides* |  | F2458 | Yellow River | MG698085 | NA | MG697459 | MG697629 |
| *T. siluroides* |  | F2459 | Yellow River | MG698086 | NA | MG697460 | MG697630 |
| *T. siluroides* |  | F2460 | Yellow River | MG698087 | NA | MG697461 | MG697631 |
| *T. siluroides* |  | LBY1 | Yellow River | NA | MN896117 | MN896384 | NA |
| *T. siluroides* |  | LBY2 | Yellow River | NA | MN896116 | MN896383 | NA |
| *T. siluroides* |  | LBY3 | Yellow River | NA | MN896115 | MN896382 | NA |
| *T. strauchii* |  | F735 | Ili River | MG698223 | KP297875 | KX373853 | MG697906 |
| *T. strauchii* |  | F736 | Junggar River | MG698224 | KP979754 | KX373854 | MG697907 |

**Table S2.** Sequence information including the GenBank accession number, species name and ID of 106 cytb sequences for divergence time estimation

| **Species** | **ID** | **River system** | **cyt *b*** |
| --- | --- | --- | --- |
| *T. robusta* | F_Y323 | Yellow River | MG697368 |
| *T. robusta* | F_Y329 | Yellow River | MG697373 |
| *T. robusta* | F_Y330 | Yellow River | MG697374 |
| *T. robusta* | F_Y3876 | Yellow River | MG697388 |
| *T. robusta* | F_Y3879 | Yellow River | MG697391 |
| *T. robusta* | F_Y3881 | Yellow River | MG697393 |
| *T. robusta* | F_Y3852 | Yellow River | MG697394 |
| *T. robusta* | F_Y3853 | Yellow River | MG697395 |
| *T. robusta* | F_Y3668 | Yellow River | MG697397 |
| *T. robusta* | F_Y3672 | Yellow River | MG697401 |
| *T. robusta* | F_Y3674 | Yellow River | MG697403 |
| *T. robusta* | F_Y2517 | Yellow River | MG697404 |
| *T. robusta* | F_Y2579 | Yellow River | MG697405 |
| *T. robusta* | F_Y2580 | Yellow River | MG697406 |
| *T. robusta* | F_Y2581 | Yellow River | MG697407 |
| *T. robusta* | F_Y3577 | Yellow River | MG697408 |
| *T. robusta* | F_Y3579 | Yellow River | MG697409 |
| *T. robusta* | F_Y2644 | Yellow River | MG697413 |
| *T. robusta* | F_Y2648 | Yellow River | MG697417 |
| *T. robusta* | F_Y2653 | Yellow River | MG697422 |
| *T. robusta* | F_Y2656 | Yellow River | MG697425 |
| *T. robusta* | F_Y2905 | Yellow River | MG697429 |
| *T. robusta* | F_Y2907 | Yellow River | MG697431 |
| *T. robusta* | F_Y3050 | Yellow River | MG697443 |
| *T. robusta* | F_J2520 | Jialing River | MG697526 |
| *T. robusta* | F_J2522 | Jialing River | MG697528 |
| *T. robusta* | F_J2523 | Jialing River | MG697529 |
| *T. robusta* | F_J2524 | Jialing River | MG697530 |
| *T. robusta* | F_J2525 | Jialing River | MG697531 |
| *T. robusta* | F_J2547 | Jialing River | MG697539 |
| *T. robusta* | F_J2532 | Jialing River | MG697544 |
| *T. robusta* | F_J3058 | Jialing River | MG697545 |
| *T. robusta* | F_J3060 | Jialing River | MG697546 |
| *T. robusta* | F_J3362 | Jialing River | MG697547 |
| *T. robusta* | F_J3363 | Jialing River | MG697548 |
| *T. robusta* | F_J3244 | Jialing River | MG697550 |
| *T. robusta* | F_J3245 | Jialing River | MG697551 |
| *T. robusta* | F_J3172 | Jialing River | MG697553 |
| *T. robusta* | F_J3184 | Jialing River | MG697565 |
| *T. robusta* | F_J3187 | Jialing River | MG697568 |
| *T. robusta* | F_J3304 | Jialing River | MG697576 |
| **Species** | **ID** | **River system** | **cyt *b*** |
| *T. robusta* | F_J3344 | Jialing River | MG697580 |
| *T. robusta* | F_J3345 | Jialing River | MG697581 |
| *T. robusta* | LZL1 | Haihe River | MN896385 |
| *T. robusta* | LZL2 | Haihe River | MN896386 |
| *T. robusta* | LZL4 | Haihe River | MN896388 |
| *T. robusta* | LZL9 | Haihe River | MN896393 |
| *T. robusta* | LBW3 | Yellow River | MN896396 |
| *T. robusta* | LBW4 | Yellow River | MN896397 |
| *T. robusta* | LBW5 | Yellow River | MN896398 |
| *T. robusta* | LCB1 | Yihe River | MN896400 |
| *T. robusta* | LCB2 | Yihe River | MN896401 |
| *T. robusta* | LCB3 | Yihe River | MN896402 |
| *T. robusta* | LCB4 | Yihe River | MN896403 |
| *T. robusta* | LCC1 | Yihe River | MN896404 |
| *T. robusta* | LCC2 | Yihe River | MN896405 |
| *T. robusta* | LCC4 | Yihe River | MN896407 |
| *T. robusta* | SXY1 | Yihe River | MN896413 |
| *T. robusta* | SXY2 | Yihe River | MN896414 |
| *T. robusta* | SXY3 | Yihe River | MN896415 |
| *T. robusta* | SXY4 | Yihe River | MN896416 |
| *T. robusta* | LNS1 | Luohe River | MN896419 |
| *T. robusta* | LNS2 | Luohe River | MN896420 |
| *T. robusta* | LNS3 | Luohe River | MN896421 |
| *T. robusta* | LNS4 | Luohe River | MN896422 |
| *T. robusta* | LSH1 | Luohe River | MN896428 |
| *T. robusta* | LSH2 | Luohe River | MN896429 |
| *T. robusta* | LSH3 | Luohe River | MN896430 |
| *T. robusta* | LSM1 | Luohe River | MN896431 |
| *T. robusta* | LSM2 | Luohe River | MN896432 |
| *T. robusta* | LSM3 | Luohe River | MN896433 |
| *T. robusta* | LSM4 | Luohe River | MN896434 |
| *T. robusta* | XA1 | Luohe River | MN896440 |
| *T. robusta* | XA2 | Luohe River | MN896441 |
| *T. robusta* | XA3 | Luohe River | MN896442 |
| *T. robusta* | XA4 | Luohe River | MN896443 |
| *T. robusta* | YYJ1 | Luohe River | MN896456 |
| *T. robusta* | YYJ2 | Luohe River | MN896457 |
| *T. robusta* | YYJ3 | Luohe River | MN896458 |
| *T. robusta* | YYJ4 | Luohe River | MN896459 |
| *T. robusta* | YYS1 | Luohe River | MN896461 |
| *T. robusta* | YYS2 | Luohe River | MN896462 |
| *T. robusta* | YYS3 | Luohe River | MN896463 |
| *T. robusta* | YYS4 | Luohe River | MN896464 |
| **Species** | **ID** | **River system** | **cyt *b*** |
| *T. robusta* | JYD1 | Luohe River | MN896470 |
| *T. robusta* | JYD10 | Luohe River | MN896471 |
| *T. robusta* | JYD2 | Luohe River | MN896472 |
| *T. robusta* | JYD3 | Luohe River | MN896473 |
| *T. robusta* | JYF1 | Yellow River | MN896480 |
| *T. robusta* | JYF2 | Yellow River | MN896481 |
| *T. robusta* | JYF3 | Yellow River | MN896482 |
| *T. robusta* | JYF5 | Yellow River | MN896484 |
| *T. robusta* | JYT2 | Yellow River | MN896492 |
| *T. robusta* | JYT3 | Yellow River | MN896493 |
| *T. robusta* | JYT4 | Yellow River | MN896494 |
| *T. robusta* | JYT10 | Yellow River | MN896483 |
| *T. robusta* | JYT11 | Yellow River | MN896487 |
| *T. robusta* | JYT12 | Yellow River | MN896477 |
| *T. siluroides* | F2458 | Yellow River | MG697459 |
| *T. siluroides* | F2459 | Yellow River | MG697460 |
| *T. siluroides* | F2460 | Yellow River | MG697461 |
| *T. siluroides* | LBY1 | Yellow River | MN896384 |
| *T. siluroides* | LBY2 | Yellow River | MN896383 |
| *T. siluroides* | LBY3 | Yellow River | MN896382 |
| *T. strauchii* | F735 | Ili River | KX373853 |
| *T. strauchii* | F736 | Junggar River | KX373854 |

**Table S3**. Sequence information including species name, GenBank accession number and sampling localities of 19 cytb for ancestral range

| **Species** | **Location** | **ID** | **River system** | **Cyt *b*** | **Locations in RASP** |
| --- | --- | --- | --- | --- | --- |
| *T. robusta* | HS1 | F323 | Yellow River | MG697368 | A |
| *T. robusta* | HS2 | F3876 | Yellow River | MG697388 | A |
| *T. robusta* | HS3 | F3852 | Yellow River | MG697394 | A |
| *T. robusta* | HS4 | F3668 | Yellow River | MG697397 | A |
| *T. robusta* | TH1 | F2517 | Yellow River | MG697404 | A |
| *T. robusta* | TH1 | F2579 | Yellow River | MG697405 | A |
| *T. robusta* | TH2 | F2644 | Yellow River | MG697413 | A |
| *T. robusta* | TH3 | F2905 | Yellow River | MG697429 | A |
| *T. robusta* | JLJ1 | F2520 | Jialing River | MG697526 | B |
| *T. robusta* | JLJ1 | F2523 | Jialing River | MG697529 | B |
| *T. robusta* | JLJ1 | F2547 | Jialing River | MG697539 | B |
| *T. robusta* | JLJ2 | F2532 | Jialing River | MG697544 | B |
| *T. robusta* | JLJ3 | F3362 | Jialing River | MG697547 | B |
| *T. robusta* | JLJ4 | F3244 | Jialing River | MG697550 | B |
| *T. robusta* | JLJ5 | F3172 | Jialing River | MG697553 | B |
| *T. robusta* | JLJ6 | F3304 | Jialing River | MG697576 | B |
| *T. robusta* | LZL | LZL1 | Haihe River | MN896385 | E |
| **Species** | **Location** | **ID** | **River system** | **Cyt *b*** | **Locations in RASP** |
| *T. robusta* | LBW | LBW3 | Yellow River | MN896396 | C |
| *T. robusta* | LCB | LCB1 | Yihe River | MN896400 | C |
| *T. robusta* | LCC | LCC1 | Yihe River | MN896404 | C |
| *T. robusta* | SXY | SXY1 | Yihe River | MN896413 | C |
| *T. robusta* | LNS | LNS1 | Luohe River | MN896419 | C |
| *T. robusta* | LSH | LSH1 | Luohe River | MN896428 | C |
| *T. robusta* | LSM | LSM1 | Luohe River | MN896431 | C |
| *T. robusta* | XA | XA1 | Luohe River | MN896440 | C |
| *T. robusta* | YYJ | YYJ1 | Luohe River | MN896456 | C |
| *T. robusta* | YYS | YYS1 | Luohe River | MN896461 | C |
| *T. robusta* | JYD | JYD1 | Yellow River | MN896470 | D |
| *T. robusta* | JYF | JYF1 | Yellow River | MN896480 | D |
| *T. robusta* | JYT | JYT2 | Yellow River | MN896492 | D |
| *T. robusta* | JYT | JYT10 | Yellow River | MN896483 | D |

**Table S4**. The 29 samples information for species distribution model of *T. robusta*

| **Species** | **Location** | **longitude** | **latitude** |
| --- | --- | --- | --- |
| *T. robusta* | HS1 | 100.881 | 37.578 |
| *T. robusta* | HS2 | 101.000 | 36.90 |
| *T. robusta* | HS3 | 101.654 | 36.977 |
| *T. robusta* | HS4 | 103.278 | 36.697 |
| *T. robusta* | TH1 | 103.022 | 35.460 |
| *T. robusta* | TH2 | 103.825 | 35.335 |
| *T. robusta* | TH3 | 104.057 | 34.473 |
| *T. robusta* | TH4 | 102.344 | 34.604 |
| *T. robusta* | JLJ1 | 103.522 | 33.973 |
| *T. robusta* | JLJ2 | 104.325 | 33.795 |
| *T. robusta* | JLJ3 | 105.080 | 33.83 |
| *T. robusta* | JLJ4 | 104.367 | 33.099 |
| *T. robusta* | JLJ5 | 104.872 | 32.849 |
| *T. robusta* | JLJ6 | 105.776 | 33.128 |
| *T. robusta* | JYT | 112.240 | 35.215 |
| *T. robusta* | JYD | 112.139 | 35.203 |
| *T. robusta* | JYF | 112.167 | 35.093 |
| *T. robusta* | LBW | 110.952 | 34.403 |
| *T. robusta* | LZL | 113.718 | 36.157 |
| *T. robusta* | LCB | 111.423 | 34.043 |
| *T. robusta* | LCC | 111.697 | 33.939 |
| *T. robusta* | SXY | 111.980 | 34.064 |
| *T. robusta* | LNS | 111.211 | 34.240 |
| *T. robusta* | XA | 112.245 | 34.758 |
| *T. robusta* | YYJ | 112.016 | 34.376 |
| *T. robusta* | YYS | 112.073 | 34.610 |
| *T. robusta* | LSM | 111.055 | 33.951 |
| *T. robusta* | LSH | 110.852 | 34.064 |
| *T. robusta* | SC | 103.670 | 33.560 |

**REFERENCES**

Feng, C. G., Zhou, W. W., Tang, Y. T., Gao, Y., Chen, J. M., Tong, C., et al. (2019). Molecular systematics of the *Triplophysa robusta* (Cobitoidea) complex: extensive gene flow in a depauperate lineage. *Mol. Phylogenet. Evol.* 132, 275–283.

Wu, H. H., Gu, Q. H., Zhou, C. J., Tang, Y. T., Husemann, M., and Meng, X. L. (2020). Molecular phylogeny and biogeography of *Triplophysa* stone loaches in the Central Chinese Mountains, *Biol. J. Linn. Soc.* 130(3), 563–577.
